# Increasing Water Absorptivity of an Aerogel-Based Coating Mortar in Subsequent Wetting and Drying

**DOI:** 10.3390/gels8120764

**Published:** 2022-11-24

**Authors:** Ali Naman Karim, Pär Johansson, Angela Sasic Kalagasidis

**Affiliations:** Department of Architecture and Civil Engineering, Chalmers University of Technology, SE-41296 Gothenburg, Sweden

**Keywords:** aerogel, coating mortar, capillary water absorption, thermal insulation, retrofitting, driving rain

## Abstract

Aerogel-based coating mortars are energy-efficient composites with thermal conductivities of 30–50 mW/(m·K). They are useful when retrofitting uninsulated building envelopes, particularly in listed masonry buildings, as shown in studies. Meanwhile, the long-term reliability of their hygrothermal properties, typically declared after a single laboratory measurement, is not confirmed. To illustrate the latter and by combining experimental and numerical methods, this study shows that (1) the capillary water absorptivity of a commercially available aerogel-based coating mortar increases after repeated drying and wetting cycles, and (2) leads to a higher moisture content in a masonry wall. After the third cycle, the measured water absorption was more than five times higher than after the first one. Based on numerical simulations, the increasing capillary water absorptivity results in 36% higher relative humidity in the wall if the aerogel-based coating mortar is applied externally and exposed to driving rain. Future research should investigate the reasons behind the observed deviations in the capillary water absorptivity and whether it applies to other types of aerogel-based coating mortars.

## 1. Introduction

Aerogel-based coating mortars (ACMs), plasters, and renders are a class of thermal insulation coating mortars with thermal conductivities around 30–50 mW/(m·K) [1]. This range also applies for conventional thermal insulation materials while it is more than 10 times lower than for conventional coating mortars. ACMs are lime and white cement-based mortars incorporating a large proportion (more than 50 vol-%) of hydrophobized aerogel granules. Other additives, such as air entraining and water-repellent agents, are added in the final mixture to get a product with the desired properties. Compared with conventional coating mortars, ACMs have higher porosity, lower density, and lower mechanical strength. Also, due to the high fraction of hydrophobic aerogel granules, they have a higher hydrophobicity as well. ACMs are vapor permeable with a vapor permeability coefficient, µ-value (−), of 4–6, which is in the lower range of most conventional coating mortars.

Because of their superior thermal insulation properties, ACMs provide new technical solutions for the energy retrofitting of uninsulated building envelopes, particularly in listed and culturally valuable masonry buildings [1,2,3]. For many of the listed buildings, restrictions related to the preservation of character-defining elements, admissible thickness of building envelopes, and existing (moisture-related) damage to the construction, reduce the number of possible retrofitting measures [1,4]. In such cases, the application of ACMs can result in increased energy efficiency and slimmer wall elements with moderate changes to the façades.

In a recent literature review we conducted [1], the state of the art of the research on ACMs was presented with the aim of identifying the existing knowledge gaps in the hygrothermal performance of ACMs. Information on ACMs was collected based on a search of the Scopus databases, Web of Science, and Google Scholar. Several combinations of keywords including ‘aerogel coating mortar’, ‘aerogel render’, and ‘aerogel plaster’ were included in the search string. The final data set included 61 scientific articles studying the hygrothermal performance of ACMs.

Previous field studies have shown that the application of 15–60 mm of ACMs on uninsulated masonry wall elements resulted in a 27–70% reduction in their U-values, confirming the high thermal insulation performance of ACMs. Meanwhile, data on the long-term hygrothermal properties of ACMs, required to perform reliable risk assessment analyses, were found to be incomplete. For the majority of commercial ACMs, moisture-dependent thermal conductivity, free-water saturation moisture content, and moisture sorption isotherm with a hysteresis effect (adsorption and desorption isotherm) are rather limited. Furthermore, the declared material properties of these ACMs are, like for other building materials, based on laboratory measurements and analyzed under controlled climatic conditions. Likewise, and apart from a number of studies in the laboratory [5,6,7], the correlation between the declared moisture properties of ACMs and their long-term performance when exposed to real climate loads in the field is not yet confirmed. This could partly be because the material is relatively new but also due to the fact that moisture safe design is practiced mainly in regions with high moisture loads on buildings [8,9,10,11,12,13]. An example of such is the south and west regions of Sweden, with subpolar oceanic climate [14], i.e., the humid temperate climate subtype (Cfb) characterized by high humidity and rain throughout the year. Reliable data on the long-term performance of ACMs in real-life applications is necessary to assess the risks and plan measures to avoid moisture-related damage in buildings. Furthermore, the relatively higher material and application cost for ACMs must be justified [1]. The material cost of a commercial ACM was declared to be around 32 €/m^2^/cm in 2020, i.e., multiple times higher than the same for conventional mortars. A more in-depth review of the cost versus performance ratio of ACMs was reported in [1].

For the declared material properties of ACMs used in risk assessment analyses, the standard EN ISO 998-1 [15] specifies the corresponding standards and laboratory-based testing methods for characterization of conventional coating mortars including thermal insulation mortars. According to Karim et al. [1], several of the laboratory-based testing methods suggested in EN ISO 998-1 have been implemented with no modifications of the methodology to characterize trial mixtures of ACMs.

As specified in EN ISO 998-1 and to estimate the capillary water absorption coefficient, A_cap_ (kg/m^2^·min^0.5^) of ACMs, the testing method stated in the standard EN ISO 1015-18 [16] is used. Accordingly, we conducted laboratory measurements following this procedure. The repeatability of the measurements was investigated when increasing water absorptivity was observed with the number of wetting and drying cycles. The observation has neither been reported nor explained previously in the literature wherein the capillary water absorptivity of ACMs and their long-term hygrothermal performance were studied.

Among the studies on ACMs, [17,18,19,20,21,22,23] reported results on A_cap_ for different trial mixtures of ACMs. Here, pre-conditioned samples were measured only once, and their properties were declared after the first wetting cycle. The range of measured A_cap_ was 0.48–2.8 kg/m^2^·min^0.5^, which is higher than the stated requirement (less than 0.4 kg/m^2^·min^0.5^) in EN ISO 998-1. Furthermore, in [5,6,7,24,25,26], the long-term performance of trial mixtures of ACMs was studied using artificial weathering cycles in the laboratory. Trial mixtures of ACMs were produced in the laboratory by mixing aerogel granules to a mortar mixture. While the majority of these studies focused on thermal performance, [6,7] also studied the water absorptivity of trial mixtures of ACMs. Maia et al. [7] evaluated the A_cap_ of ACM samples before and after they were exposed to various consecutive weathering cycles (wetting-drying, heating-freezing combined with infrared radiation) in the laboratory. The A_cap_ of the samples was measured according to EN ISO 1015-18 [16]. The measured A_cap_ was around 0.8 kg/m^2^·min^0.5^ [7] before and after aging, i.e., stable, although very high, for a thermal insulation coating mortar. In another study done by Sakiyama et al. [6], large-scale wall prototypes of trial mixtures of ACM were exposed to severe weathering cycles including combinations of heating, cooling, and rain loads in the laboratory. The walls insulated by ACM showed signs of excessive water intrusion after weathering cycles in [6]. In both studies [6,7], the importance of correct choice of base coat and protective coating was highlighted to avoid excessive water intrusion in the material.

This study further investigates the impact of repeated drying and wetting cycles on the A_cap_ of a commercial ACM. A set of measurements consisting of three repeated capillary water absorption tests (three wetting and drying cycles) were performed in accordance with EN ISO 1015-18 [16] with a moderate upscaling of the sample size, see Section 2.1 and Section 4.1. Furthermore, the influence of the varying capillary water absorptivity on the long-term hygrothermal performance of ACMs in practice was explored using numerical simulations. The material will likely be exposed to rain and rainwater leakage in real-life applications. A case study was therefore carried out in a listed and moisture-damaged church in Sweden.

The results of the laboratory measurements and numerical hygrothermal simulations are presented in Section 2.1 and Section 2.2, respectively. To enhance the readability, Section 2.2 includes only the most relevant graphical results with respect to the scope of the numerical simulations. Other relevant graphical results are compiled in Appendix A, Appendix B, Appendix C and Appendix D. In Section 3, the conclusions of the conducted studies are compiled, and in Section 4, the material and methods of the studies are described in detail.

## 2. Results and Discussion

In this chapter, the results of the conducted studies are presented and discussed. First, the results of the experimental studies, carried out in a laboratory, are presented in Section 2.1. Second, the results of the parametric studies using numerical hygrothermal simulations are presented in Section 2.2. While the full methodology is provided later in Section 4, only an excerpt is shown in this section to help the reader understand the results.

### 2.1. Laboratory Measurements

**Figure 1:**
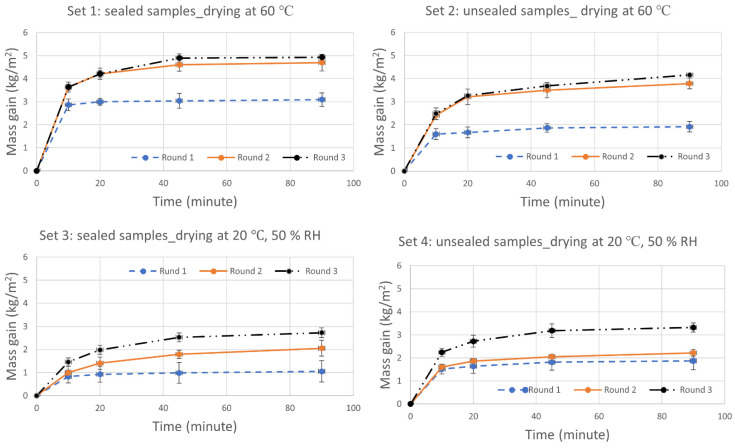
The mass gain (kg/m^2^) of all sample sets (1–4) during 90 min of capillary water absorption from free water. Each measurement point represents the mean value of the three samples in each set.

**Figure 2:**
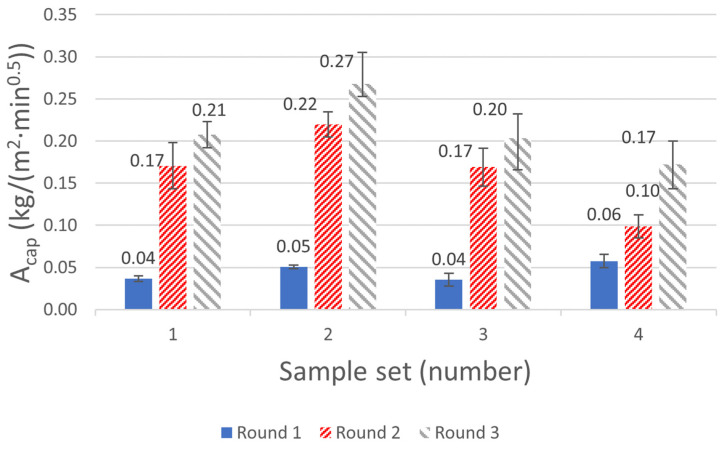
The calculated water absorption coefficient, A_cap_ (kg/m^2^·min^0.5^), for all three rounds of measurement using Equation (1). Each value represents the mean value of three samples in each set. The declared A_cap_ of the ACM is stated to be less than 0.2 kg/m^2^·min^0.5^.

In total, four sample sets of three ACM samples each were considered for the measurement of A_cap_ following the standard EN ISO 1015-18 [16]. Each set was exposed to three wetting and drying cycles. The impact of the selected drying temperature and sealing of the samples was also evaluated in the measurements. The samples were either sealed or unsealed on the side edges and dried either at elevated or ambient temperature (T). Figure 1 and Figure 2 show the water mass gain of the samples (kg/m^2^) for 90 min of contact with free water, and the corresponding calculated A_cap_ (based on Equation (1)), respectively. Each measurement point represents the average value of the three samples in the considered sample set. The standard deviation (SD = $\sqrt{\frac{\sum_{i=1}^{\mathrm{number}\;\mathrm{of}\;\mathrm{samples}}{\left(\mathrm{mean}\;\mathrm{value}-{\;\mathrm{measured}\;\mathrm{value}}_{i}\right)}^{2}}{\mathrm{number}\;\mathrm{of}\;\mathrm{samples}-1}}$) (SD) for each measurement point is illustrated in Figure 1 and Figure 2.

For the calculated A_cap_ presented in Figure 2, it has to be mentioned that according to the standard [16], the A_cap_ should be calculated for the samples dried at elevated temperature (60 °C). As such, the calculated values for sample sets 2 and 4 (dried at 20 °C and 50% RH, respectively) are not following this specification. Consequently, for these two sample sets, it is the trend and actual change in the capillary water absorptivity after each cycling that is of interest rather than the absolute value of the calculated A_cap_. With this reasoning in mind, all sample sets are considered in the analyses.

The results showed that the calculated A_cap_ in the first round was 0.04–0.06 kg/m^2^·min^0.5^ for all sample sets, i.e., within the lower range of the declared value of less than 0.2 kg/m^2^·min^0.5^. The value for sample set 1, dried and sealed in accordance with the standard, was 0.04 kg/m^2^·min^0.5^. In the second and third round of the measurements, the A_cap_ for the same sample set 1 was significantly increased from 0.04 to 0.17 and 0.21 kg/m^2^·min^0.5^, respectively. This means that the measured A_cap_ in the third round was more than five times higher than the same after the first round. A similar magnitude of change could be observed for the other sample sets as well, see Figure 2.

As mentioned in Section 4.1, an additional fourth round of measurements was also carried out for two out of three samples in set 3, which is not included in the presented results. Like the previous round of measurements, the total mass gain for 90 min was increased and the new A_cap_ was calculated as 0.25 kg/m^2^·min^0.5^. This corresponds to a 22% higher value than the same from the third round. Based on the experimental results presented in this section, it is evident that the mass gain and measured A_cap_ of the ACM samples already increased significantly after one cycle of wetting and drying. The drying condition and sealing of the samples were investigated as two parameters that could potentially affect the measurements. Regardless of the sealing and drying condition, a similar increasing trend was observed in the measurements. For the unsealed samples, a source of error would be the additional vapor exchange via diffusion with the (humid) surrounding air in the containers. The coefficient of variance (CV = $\left(\frac{\mathrm{SD}}{\mathrm{mean}\;\mathrm{value}}\right)\cdot 100$) (CV) for the A_cap_ of the sample sets varied between 7–22%. These variations could be due to the uncertainties related to the testing method, such as weighting of the samples. It could also be a sign of heterogeneity of the samples with respect to their capillary water absorptivity.

The increasing A_cap_ of ACMs has not been reported earlier and thus no unequivocal explanation for this unexpected observation could be found in the literature. Additional literature on similar materials were reviewed to investigate the basis for this phenomenon. No studies could be found that discuss the presented observations. On the contrary, reduced water absorptivity has been reported earlier for concrete samples exposed to repeated wetting and drying cycles [27].

A possible explanation for the increasing A_cap_ of the ACM samples could be a structural change or damage, such as micro cracking, introduced internally in the samples due to shrinkage strains and stresses caused by the wetting and drying cycles. In such a case, the results indicate that only one wetting and drying cycle at ambient temperature would be sufficient to cause such structural damage in the material affecting its capillary water absorptivity. This could in turn be possibly motivated by the low mechanical strength of ACMs compared with other mortars. A second possible explanation could relate to the hydrophobicity of the ACMs and aerogel granules that could alternatively be partly deteriorated because of the wetting and drying cycles. However, one could expect higher stresses than only one cycling to cause such level of deterioration. Additionally, the study presented by Stahl et al. [2] showed that hydrophobized aerogel granules could resist harsh and alkali environments for longer periods without any sign of significant damage due to water intrusion inside the aerogel granules. As for the third explanation, the observations could also connect to the pore structure and moisture distribution in the samples before and after each wetting and drying cycle. In porous materials, the moisture distribution can be inhomogeneous. Once the material is exposed to wetting and later drying, the moisture distribution in the material may vary from the original distribution. Consequently, this could result in different moisture flow paths in the material potentially explaining higher moisture absorption during a specific time, as in the case of the measurements presented. The proposed explanations have not yet been investigated. Thus, further research will be required to investigate the reasons behind the observed increasing A_cap_ of the ACM samples, and whether this observation is product specific or if it applies to all ACM products.

The declared values of A_cap_, used in hygrothermal risk assessment analyses, are based on laboratory measurements similar to the first-round measurements in this paper. Questions can thus be raised on the reliability of the declared values for ACMs considering their long-term performance in practical applications. As described in Section 4.2, it should be kept in mind that ACMs are in practice applied in a multilayer wall system and thus less directly exposed to free water absorption compared with the laboratory measurements. However, long term, this could take place in the case of damage and rainwater leakages in the construction.

### 2.2. Numerical Hygrothermal Simulations

In the numerical analyses performed, three scenarios and three wall configurations were defined. Wall A represented the reference brick wall, while wall B represented an internally insulated wall with ACM. Additionally, wall C was defined as representing an external insulation of the brick wall (wall A) with ACM. For the wall configuration B and C, three ACMs with different A_cap_ were defined (B1–B3 and C1–C3). In this section, the result of the simulations in each scenario is presented and discussed. The presented results correspond to the last year (out of 5) of simulations. To ease the readability, only the most relevant results and graphical materials with respect to the scope of the considered scenarios are presented in Section 2.2. Additional relevant graphical materials are compiled in the Appendix A, Appendix B, Appendix C and Appendix D.

**Scenario 1**

**Figure 3:**
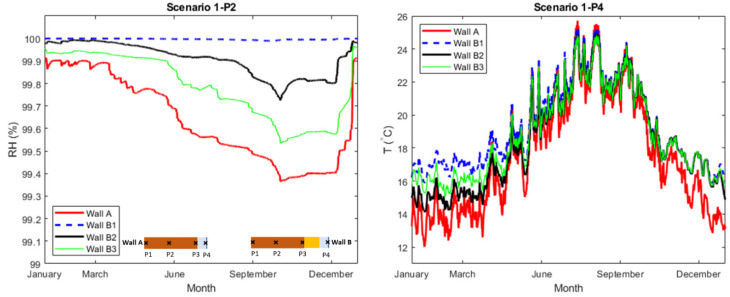
(**Left**): Relative humidity at checkpoints P2 (middle of brick). (**Right**): Temperature at checkpoint P4 (interior surface).

In scenario 1, the exterior wall surfaces were exposed to rain (Adhering Fraction of Rain, AFR: 0.7) and the drying towards the exterior was highly limited, simulating a case for the locally denser and low permeable bricks in the moisture-damaged reference building. In Figure 3, the RH at checkpoint P2, in the middle of the brick, and the interior surface T at point P4 are plotted. Table 1 presents the yearly average RH at all checkpoints. Furthermore, additional plots on scenario 1 can be found in Appendix B (Figure A3 and Figure A4).

In summary, the simulated cases in scenario 1 showed high levels of RH in the walls, both for the reference wall but also the walls internally insulated with ACM. The high RH-levels in the reference wall, wall A, could demonstrate an extreme case of the moisture problems that exist today in the walls of the reference building. In the case the drying towards exterior is limited, the interior drying may not be sufficient even for the heated indoor environment as demonstrated in the simulations. The results also showed that for the rain-exposed walls, the existing moisture problems would remain after internal insulation by ACM if drying towards exterior is prevented. The differences between the wall B1 and wall B2–B3 were negligible considering the RH-levels at checkpoints P1–P3. At checkpoint P4 at the interior, the average RH in wall B1 (A_cap_: 0.04 kg/m^2^·min^0.5^) was 21% lower than the same in wall B2 (A_cap_: 0.2 kg/m^2^·min^0.5^) and wall B3 (A_cap_: 0.3 kg/m^2^·min^0.5^). Comparing the interior surface temperature at P4, Figure 3 (right), the average temperature was around 17.6 °C in wall A and 19.2 °C in wall B1. The same in wall B2–B3 was around 18.5 °C. This could be an indicator for less transmission heat losses towards the exterior and better thermal comfort in the interior for the retrofitted walls B1–B3. The higher temperature at P4 in wall B1 compared with wall B2–B3 could be due to the lower moisture content and accordingly lower thermal conductivity of the ACM in wall B1. Considering the risk for freeze-thawing, it only occurred at the exterior of the brick section (P1). A freeze-thaw cycle was defined as one crossing of 0 °C in the respective checkpoint. The number of freeze-thaw cycles was increased from 21 to 25 times (Figure A4) after internal insulation by ACM. Again, this was most likely a consequence of reduced heat losses towards the exterior resulting in lower temperatures in the exterior (P1).

**Scenario 2**

**Figure 4:**
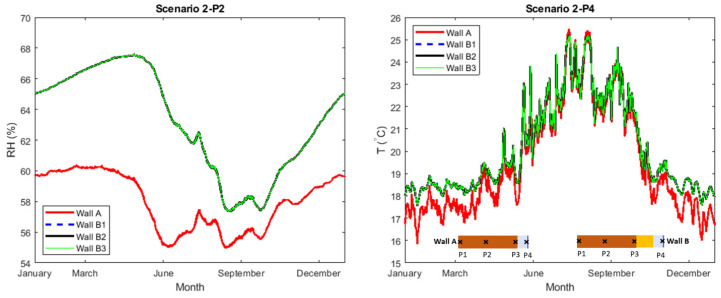
(**Left**): Relative humidity at checkpoints P2 (middle of brick). (**Right**): Temperature at checkpoint P4 (interior surface).

The simulations in scenario 2, represented the same cases as in scenario 1 with the only difference of assuming no capillary water absorption (no rain) at the exterior of the brick walls (AFR: 0). In Figure 4, the calculated relative humidity at checkpoint P2, in the middle of the brick, and the interior surface temperature at point P4 are plotted. Table 2 presents the yearly average RH at all checkpoints. Furthermore, additional plots in scenario 2 can be found in Appendix C (Figure A5 and Figure A6).

In general, and compared to scenario 1, the RH-levels in the walls simulated in scenario 2 were lower. Like scenario 1, a less significant difference between the calculated RH-levels in the walls was observed. The results showed that freeze-thaw cycles occurred only at the exterior part of the brick section (Figure A6), as in scenario 1. The number of cycles at checkpoint P1 was 35 in wall A compared to 41–42 for wall B1–B3. Compared with scenario 1, the number of cycles was increased in scenario 2. Unlike the wet construction and wet ACM in scenario 1, the thermal performance of the dryer construction in scenario 2 would be higher. As such, the heat losses towards the exterior would be less, increasing the probability for minus degrees at the exterior. Considering the interior surface temperatures at checkpoint P4, the average temperature of the reference wall (wall A) was 19.6 °C. The temperature at the same position was increased to 20.2 °C after the internal insulation by ACM (wall B1–B3).

The results of the simulations in scenario 1 and scenario 2 illustrated that the impact of varying A_cap_ of the ACM had a limited impact on the RH-levels in the walls when ACM was applied internally. The results also showed that internal insulation with ACM could be a justified solution to prevent rainwater intrusion on the exterior of the reference building (scenario 2). The latter result is also in line with previous research [28,29] on internal application of ACM, highlighting the importance of the exterior surface conditions to reduce the water intrusion into the construction.

**Scenario 3**

**Figure 5:**
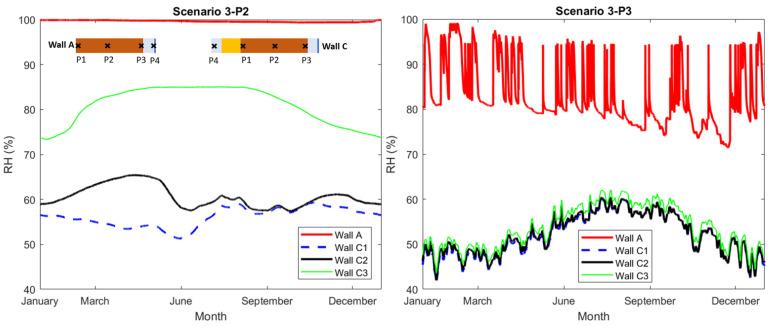
(**Left**): Relative humidity at checkpoint P2 (middle of brick). (**Right**): Relative humidity at checkpoint P3 (interior of the brick).

In scenario 3, in contrast to scenario 1 and 2, the reference wall was insulated externally by ACM. Similar to scenario 1, the exterior surface was exposed to rainwater (AFR:0.7). The purpose of the simulations in scenario 3 was to study the impact of varying A_cap_ of ACM on the RH-levels in the walls when the ACM is applied externally. In Figure 5, the calculated RH at checkpoint P2, in the middle of the brick, and at point P3, at the interior side of the brick, are plotted. Table 3 presents the yearly average RH at all checkpoints. Furthermore, additional plots in scenario 3 can be found in Appendix D (Figure A7).

Based on the results in scenario 3, the varying A_cap_ of ACM had a major impact on the RH-levels in the walls in the case of external insulation by ACM. The difference between the average RH-levels in the brick section of wall C1, with the lowest A_cap_, and the other two walls, C2 and C3, was up to 36%. This variation could in some cases be a major uncertainty with noticeable impact on the performed analyses. The simulated walls in scenario 3 were exposed to rainwater on the exterior. If the capillary water absorption at the exterior surface were to be limited, one could expect that the impact of the varying A_cap_ of ACM would be less significant than the case presented in scenario 3. In such a case, water could still reach the layer of ACM if any damage or water leakage in the construction occurred. This could in turn expose the ACM to similar wetting and drying cycles as the ones implemented in the laboratory measurements presented in Section 4.1, affecting the A_cap_ of ACM.

## 3. Conclusions

In this paper, laboratory-based measurements were carried out to measure the capillary water absorptivity, A_cap_, of a commercial aerogel-based coating mortar exposed to three cycles of wetting and drying. The results showed that the measured values increased repeatedly after each cycling. The measured values after the third cycle were more than five times higher than the initially measured values. In addition to the laboratory measurements, numerical hygrothermal simulations were used to evaluate the impact of the measured deviations in the capillary water absorptivity of the aerogel-based coating mortar on the long-term performance in the field. A moisture-damaged listed church situated in Gothenburg, Sweden was used as the reference building. Based on the simulations, the varying capillary water absorptivity of the coating mortar had a limited impact on the relative humidity levels in the construction when applied internally. Meanwhile, for external application, the impact of increasing capillary water absorptivity of the material was significant, if exposed to rain. This could in turn lead to a potential underestimation of the relative humidity levels in the construction. Future research will be required to investigate the reasons behind the observed deviations in the capillary water absorptivity and whether this phenomenon applies to all aerogel-based coating mortars.

## 4. Materials and Methods

In this chapter, the details of the conducted studies are described. Section 4.1 deals with the experimental studies in the laboratory and Section 4.2 describes the parametric case study using numerical hygrothermal simulations.

### 4.1. Laboratory Measurements

As mentioned in Section 1, EN ISO 998-1 [15] defines the specific standards that must be followed for the characterization of all types of coating mortars. For the determination of A_cap_ (kg/m^2^·min^0.5^), the standard testing method described in EN ISO 1015-18 [16] is specified. Accordingly, the A_cap_ of ACM samples was measured in a laboratory based on the EN ISO 1015-18 standard [16]. In the following, the key details of the measurement conditions specified in the standard [16] are summarized.

For the measurements, three prisms (160 mm × 40 mm × 40 mm) of the studied mortar should be casted and cured for 28 days [16]. The temperature and relative humidity conditions during the first seven days is specified at 20 ± 2 °C, 95 ± 5% RH, and the remaining 21 days 20 ± 2 °C and 65 ± 5% RH. At the end of the curing period, the four long sides (160 mm × 40 mm) of the prisms need to be sealed before the prisms are broken into two halves (80 mm × 40 mm × 40 mm). The produced half prisms are the specimens used in the measurements. Next, the samples are dried in a ventilated oven at a temperature of 60 ± 5 °C until constant mass (less than 2% of mass loss between two subsequent weightings with a 24 h interval). The samples are later placed in a tray (minimum depth of 20 mm) and immersed in water (5–10 mm) for 90 min, and weighting of the samples is carried out after 10 and 90 min from the start. Throughout the measurements, evaporation from the wet specimens should be prevented by covering the tray, and continuous surface contact with water should be maintained. The A_cap_ (kg/m^2^·min^0.5^) of the samples is calculated by their registered weights (gram) at 10 and 90 min (M1 and M2, respectively), using Equation (1).

A_cap_ = 0.1·(M2 − M1) (1)

Currently, there are less than 10 commercialized ACM products in the European market [1], of which only a few are in the stage of large-scale production. In this study, a commercial ACM that could potentially be of interest for future application in the Swedish building industry was considered. The selected ACM was composed of a hydrated lime and white cement-based binder, aerogel granules, and mineral lightweight aggregates. The declared material properties of the ACM, collected from the Technical Data Sheet (TDS) of the product [30], are presented in Table 4. The declared A_cap_ of the ACM, relevant for this study, is stated to be less than 0.2 kg/m^2^·min^0.5^, measured according to the standard [16].

In total, four sets of ACM samples were prepared following the procedure stated in ISO EN 1015-18 [16]. The mixing of the fresh mortar was based on the mixing recipe stated in the TDS of the product.

**Figure 6:**
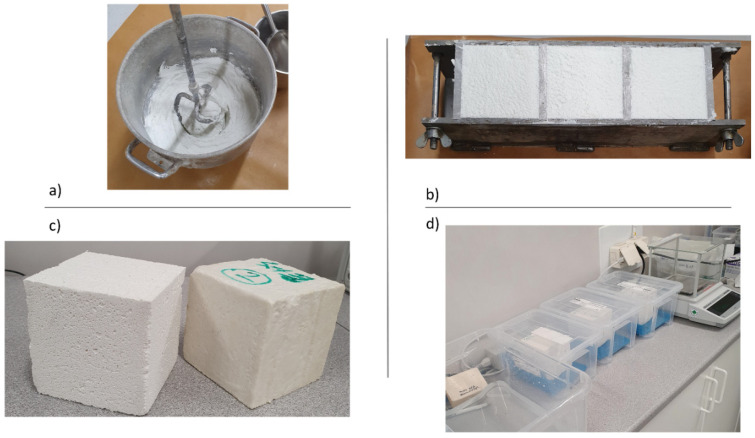
(**a**,**b**) Mixing and casting of the cubic samples. (**c**) Hardened sample: unsealed (left) and sealed on the edges by epoxy (right). (**d**) The set up used for the measurements. Continuous surface contact with water was maintained in all containers (minimum water level of 5–10 mm). A high absorbent dishcloth (blue) was placed at the bottom of each container to help maintain constant and even water content on the entire surface area of each sample. Photo: the authors.

Table 5, compiles the sample details and testing conditions implemented. Each sample set consisted of three identical samples, see Figure 6a–c. For each set, three rounds of testing were performed, i.e., three wetting and drying cycles. For sample set 3, an additional fourth round was also carried out for two out of three samples as the third sample was damaged. However, only the results of this additional fourth round are part of the discussion. After each testing round, the tested samples were dried back to the starting weight before a new round of testing was initiated. Sample sets 1 and 2 were dried at an elevated temperature (60 °C), according to the standard [16]. The other two sample sets (set 3 and 4) were dried at ambient climate (20 °C, 50% RH). For two sets (set 1 and 3), the edges of the sample were sealed (Figure 6c) while the other two sets were kept unsealed. These variations among the sample sets were to consider the impact of the drying condition and sealing of the samples on the measurement results. Figure 6d depicts an example of the considered measurement set up. During testing, the samples were kept in closed containers to avoid undesired evaporation into the surroundings. The water in the containers was maintained at the same level (minimum of 5–10 mm). Also, a highly absorbent dishcloth (Wettex) shown in blue in Figure 6d was placed at the bottom of each container to help maintain constant and even water contact with the entire surface area of each sample. The mass gain of the samples was registered after 10, 20, 45 and 90 min, respectively. A scale of the model METTLER TOLEDO PG503-S [31] with a resolution of 0.001 g was used.

As shown in Table 5, initial attempts were made to prepare prismatic samples as suggested by the standard [16]. However, challenges associated with difficulties properly cutting and sealing the hardened prisms resulted in a need to use larger samples. This decision was taken since the results differed significantly from the conventional mortars that the standard test conditions are designed for. Among others, and as pointed out in Section 1, ACMs have much lower density and mechanical strength, and higher hydrophobicity compared with conventional mortars. Instead of (half) prisms (40 mm × 40 mm × 80 mm), cubic samples (100 mm × 100 mm × 100 mm) were prepared and used for the measurements. The larger cubic samples were more easily processed after hardening, and the sample cutting could be avoided. Additionally, due to this upscaling and the increased sample mass, the requirement of less than 2% mass loss for the two subsequent weightings during drying [16] was easier to ensure (0.36 g for a cubic sample and 0.05 g for a half prism). Meanwhile, the larger cubic samples would require a longer drying period between the testing rounds. Because of a larger surface area for cubic samples (0.01 m^2^) compared to prismatic ones (0.0016 m^2^), the impact of boundary effects (water absorption via sample edges and corners) on the measurement results could also be considered as less. When using Equation (1) for estimation of A_cap_, the measured mass (M2 and M1) was recalculated to correspond to the same as that of the prismatic sample (with a surface area of 40 mm × 40 mm), which the expression in Equation (1) is based on. The recalculation of the mass was done to consider the upscaling of the sample size assuming a linear relation between the contact surface area and mass gain. This assumption, in relation to the constant coefficient (0.1) used in Equation (1), has not been confirmed by experimental studies.

### 4.2. Impact Case Study on a Reference Building: Numerical Hygrothermal Simulations

Numerical hygrothermal simulations were used to assess the impact of varying capillary water absorptivity of the ACM on the hygrothermal performance in a real-life building application. The varying A_cap_ of ACM was selected with respect to the observations made in the laboratory measurements. For this study, a moisture-damaged listed building in Gothenburg, Sweden was selected as the reference building to be retrofitted by ACM. In Section 4.2.1, the reference building is introduced. In Section 4.2.2, a general description of the multilayer wall system with ACM, i.e., how the ACM is applied in practice, is presented. Finally, in Section 4.2.3, the specifications made in the numerical hygrothermal simulations, i.e., the different simulation scenarios and wall configurations are revealed.

#### 4.2.1. Reference Building: Örgryte New Church

**Figure 7:**
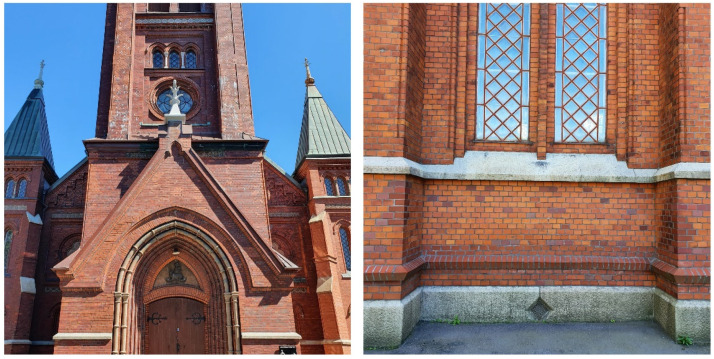
Parts of the exterior of the Örgryte New Church, selected as a reference building in this study. Photo: the authors.

The Örgryte New Church is a listed property from 1890 situated in Gothenburg, on the west coast side of Sweden. This church, located on a hill in Örgryte parish, is oriented with the main tower facing southwest [32]. The façade of the church consists of machine-made red bricks with decorative details in concrete and limestone. Figure 7 shows parts of the exterior of the church.

**Figure 8:**
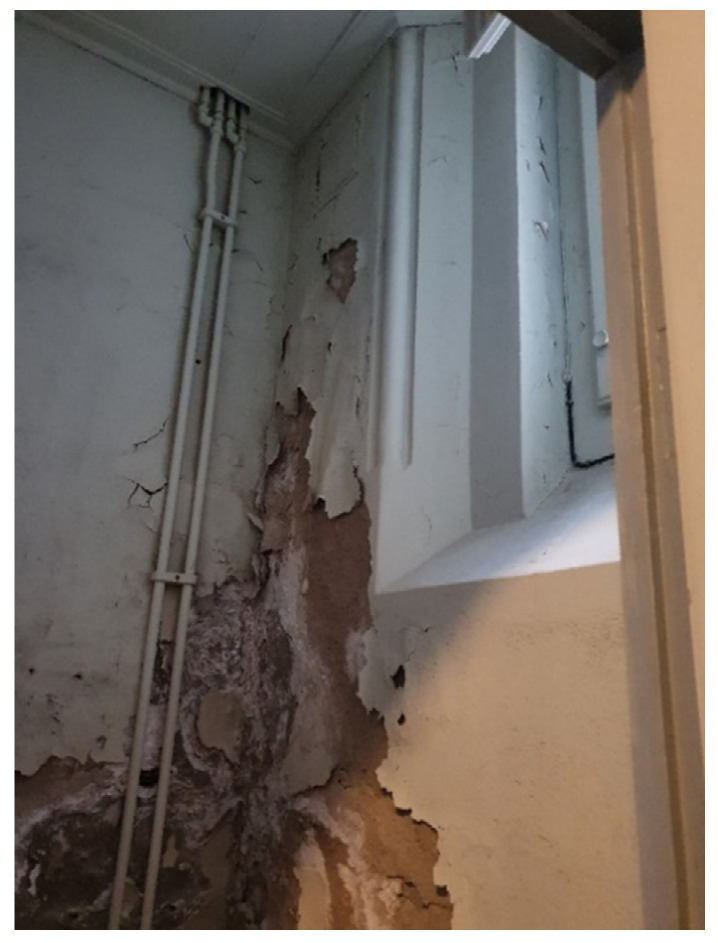
Example of the moisture-related damage at the interior of the Örgryte New Church: weathering of the internal coating mortar (plaster) and paint, and salt efflorescence. Photo: the authors.

There have been multiple cases of moisture-related damage in the church for years [32]. In [32], Balksten et al. delivered a comprehensive investigation report on the historical background, current status, and main reasons causing this damage. The first damage was reported in early 1900, approximately 10–20 years after the inauguration of the church. Since then, several retrofitting attempts have taken place to repair only limited parts of the damaged construction, most likely due to economical limitations and limited resources at the time. Consequently, the façades consist today of several types of bricks with various properties considering their vapor permeability. Also, many grout types, with varying age, binder type and porosity, and thus different moisture properties have been used for the repair work of the construction. According to Balksten et al. [32], the low frost resistance of the originally used bricks was a major contributor to the frost damage in the façades. The issue was more severe for those parts facing south and west, due to higher exposure to driving rain combined with the high frequency of freeze-thawing events. Also, the façades of the unheated areas in the church were more damaged than other parts with an internally heated area. The damage has led to weathering of the bricks from the façades with extensive material loss. Internally, the damage can be recognized locally on the weathered coating layers (plaster). Moreover, clear patterns of salt efflorescence can be observed. Figure 8 shows an example of the damage on the interior side of the church. The walls in the church were internally painted with several layers of organic and dense paints, with low vapor permeability. Consequently, drying towards the interior was rather limited. This issue resulted in moisture accumulation and salt crystallization behind the paint as the salt transportation, in dissolved form, towards the interior surface was highly prevented.

According to [32], the main moisture source for the observed damage in the building envelope of the church is the exterior moisture load mainly from driving rain. The material variations in the exterior façades, combined with high exposure to driving rain and freeze-thawing have resulted in water penetration in several spots. Meanwhile, for the parts with less permeable bricks drying towards the exterior is rather limited. The fact that drying towards the interior was also partly prevented due to the low vapor permeable interior surface coating and paint, increased the magnitude of the observed damage in the building envelope. These issues have been concluded to be the main reasons for the damage that have occurred in the building envelopes of the church.

According to the property manager of the church (E. Forseström, personal communication, 9 December 2021), the church also has other problems such as insufficient thermal comfort, high indoor air humidity, and low indoor air temperature, as well as high energy use compared with other similar churches in the surrounding area. By retrofitting the building envelope of the church using ACM, a contribution can be made to resolve some of these existing challenges. Due to the limitations related to the preservation of character-defining elements, the ACM can only be applied internally. The existing coating and paint can be removed and replaced by a multilayer wall system with ACM.

#### 4.2.2. Multilayer Wall System with ACM

**Figure 9:**
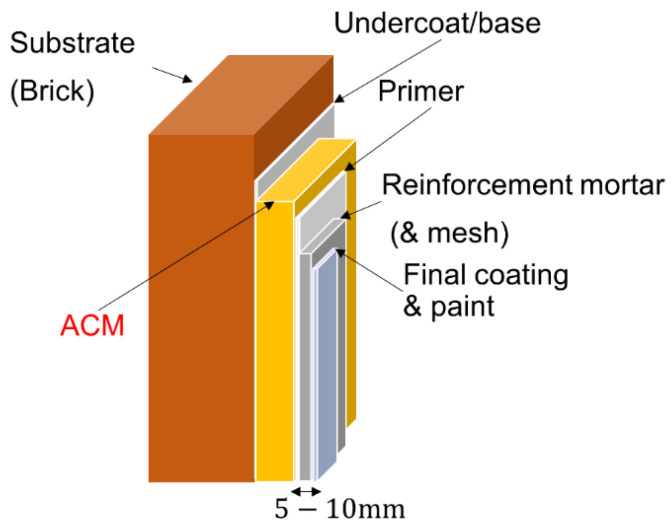
Schematic illustrating a multilayer wall system with ACM. To compensate for the low mechanical strength of the ACM, it is applied in a multilayer wall system. On the load-bearing structure, an undercoat layer is applied under the ACM (10–50 mm). To protect the ACM, a primer, reinforcement mortar and layer of coating and paint is applied to the surface. In total, this led to the system being 5–10 mm thicker than the ACM layer.

To compensate for the lower mechanical strength and higher fragility of ACM, it is in practice applied in a multilayer wall system [1], see Figure 9. Each layer of the system is applied separately and dried out before the application of the next one. An undercoat is applied first to increase the adhesiveness and control the water suction to the substrate before the ACM is applied. The thickness of the ACM is normally around 10–50 mm. On top of the ACM, a primer is applied before the application of the reinforcement mortar that provides the necessary mechanical strength to the wall system. In some cases, reinforcement mesh can also be added. Finally, the multilayer wall system can be supplemented by a final layer of coating and a mineral-based vapor open and water-repellent paint to achieve the final desired surface structure and appearance. The total thickness of the wall system is normally increased by 5–10 mm due to the additional layers applied together with ACM.

#### 4.2.3. Numerical Hygrothermal Simulations

For the numerical analyses in this paper, the software WUFI [33], version 3.4 was used. WUFI is recognized software for building physics applications and it has been used and verified in several previous studies to solve coupled one- and two-dimensional heat and moisture transport in multilayer wall systems with ACMs [3,21,23,29]. In Equations (2) and (3), the governing equations for heat and moisture transport are given:

(2) $$\frac{\partial H}{\partial T}\frac{\partial T}{\partial t}=\nabla \cdot \left(\lambda \cdot \nabla T\right)+h_{v}\cdot \nabla \cdot \left(\delta_{p}\nabla \left(RH\cdot P_{sat}\right)\right)$$

(3) $$\frac{\partial W}{\partial RH}\frac{\partial RH}{\partial t}=\nabla \cdot \left(D_{RH}\cdot \nabla RH+\delta_{p}\cdot \nabla \left(RHP_{sat}\right)\right)$$

where *H* (J) is enthalpy, *T* (°C) is temperature, *λ* (W/(m·K)) is thermal conductivity, $h_{v}$ (J) is the evaporation enthalpy of water, $\delta_{p}$ (kg/m·s·Pa) is water vapor permeability, RH (−) is relative humidity, $P_{sat}$ (Pa) is saturation vapor pressure, W (kg/m^3^) is moisture content, and $D_{RH}$ (kg/m·s) is the liquid conduction coefficient.

The numerical analyses in WUFI utilizes the finite volume technique and implicit scheme for the discretization in space and time, respectively [34]. The driving potential for heat and moisture transports through the materials are defined by temperature and relative humidity. The liquid water transport in the construction is calculated based on the moisture content and liquid water transport coefficient for suction, *D_ws_* (m^2^/s) and for redistribution *D_ww_* (m^2^/s) [34]. In simple terms, *D_ws_* describes the capillary water uptake in case of rain while D_ww_ describes the moisture redistribution when there is no rain. Because the redistribution occurs mainly in the smaller capillaries, with higher flow resistance compared with the larger ones, the process of redistribution is slower than suction. Accordingly, the *D_ww_* is normally several times smaller than *D_ws_*. The measured values of *D_ws_* and *D_ww_* are available only for a limited number of materials. For this reason, if the corresponding values are missing for a material, WUFI implements a simplified approximation method to estimate these values based on the A_cap_ (kg/m^2^·s^0.5^), W (kg/m^3^), and free water saturation $W_{f}$ (kg/m^3^) of the considered material. Equation (4) shows the expression used in WUFI to estimate *D_ws_* (m^2^/s).

(4) $$D_{ws}=3.8\cdot {\left(\frac{A_{\mathrm{cap}}}{w_{f}}\right)}^{2}\cdot 1000^{\frac{W}{W_{f}}-1}$$

In WUFI, the fraction of rainwater hitting the façades and available for capillary water absorption is specified by the unitless parameter Adhering Fraction of Rain (AFR). In the case all rainwater is available for absorption, the value is set to 1. If no capillary absorption occurs, the value is set to 0. WUFI recommends a value of 0.7 for moderately exposed façades [33].

**Simulated scenarios and wall elements**

**Figure 10:**
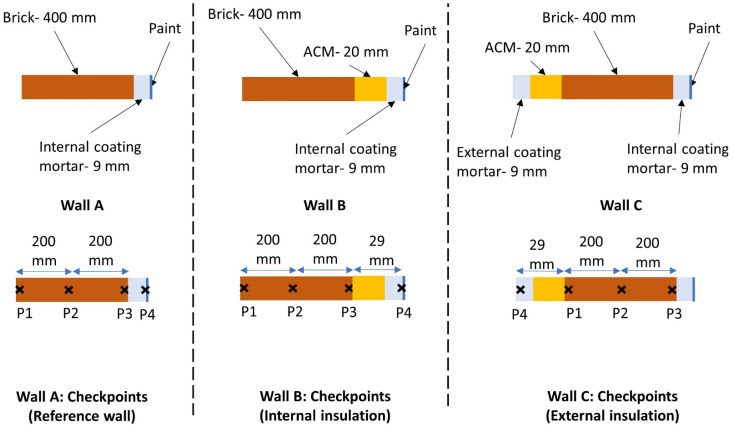
Geometries of the simulated wall elements (wall A, B, and C) and the positioning of the checkpoints (marked with ×).

In total, three wall configurations (wall A, wall B, and wall C) were considered in the simulations. The hygrothermal condition of these walls was studied for different simulation scenarios and at the checkpoints marked in Figure 10. Wall A represented the existing brick wall in the reference building where the problematic interior coating and paint have been removed and replaced by a new layer of coating and a vapor open paint. In wall B, the same brick wall was instead insulated internally by a multilayer wall system with 20 mm of ACM. Finally, wall C represented a case where the brick wall was insulated externally by a multilayer wall system with 20 mm of ACM. Like wall A, the original interior coating and paint were removed and replaced by new ones. As mentioned in Section 4.2.1, an external insulation of the reference building by ACM is not permitted in real-life practice. Thus, the case represented by wall C is only of theoretical value for the sake of comparison.

For simplicity, the analyses were conducted by one-dimensional (1D) simulations and the brick wall was considered as a homogeneous layer. To find the proper initial conditions in the wall, five consecutive years were simulated, while results were presented for the last year only. As presented in Section 4.2.1, the existing brick walls of the reference building consist locally of denser brick elements with lower vapor permeability at the exterior, limiting the vapor transport towards the outdoor. To take this into account in the simulations, as the material properties of the bricks were unknown, an additional vapor resistance with an sd-value of 0.1 m (a resistance corresponding to 0.1-m-thick layer of stagnant air) was defined on the exterior side of the brick walls. For comparison, this resistance corresponds to one-fifth of a vapor retarder (minimum sd-value: 0.5 m [35]) on the exterior side of the wall. Thus, the simulations represented a rather extreme case where the drying towards the exterior was limited. This conservative approximation has not been confirmed by experimental studies.

**Figure 11:**
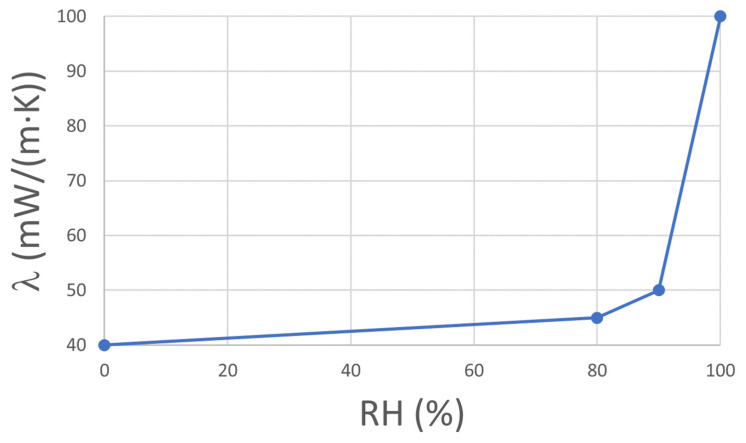
Declared moisture-dependent thermal conductivity of the ACM. The thermal conductivity is increased by less than 13% up to 80% RH and then rises sharply up to 100 mW/(m·K) at saturation [30].

**Figure A1:**
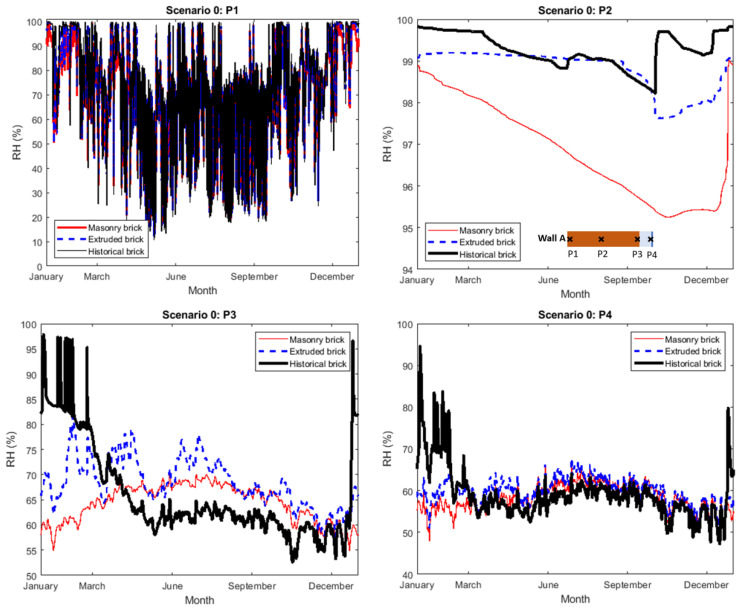
Scenario 0: Relative humidity at checkpoints P1–P4.

**Figure A2:**
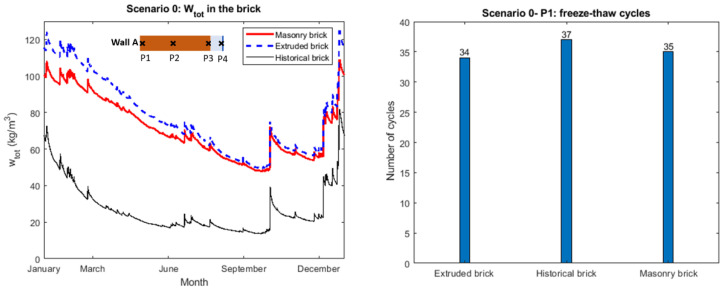
Scenario 0: Total water content in wall A and number of freeze-thaw cycles at checkpoint P1 (exterior surface).

**Figure A3:**
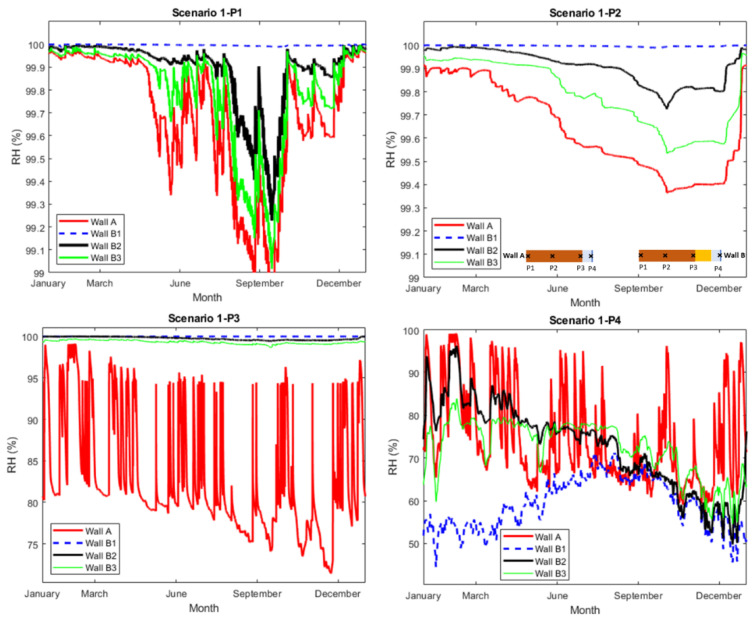
Scenario 1: Relative humidity at checkpoints P1–P4.

**Figure A4:**
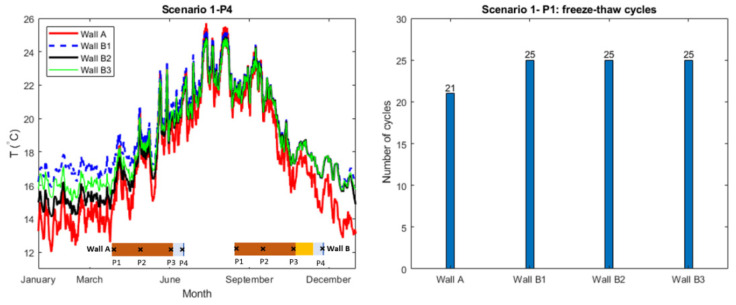
Scenario 1: Temperature at checkpoints P4 and number of freeze-thaw cycles at checkpoint P1.

**Figure A5:**
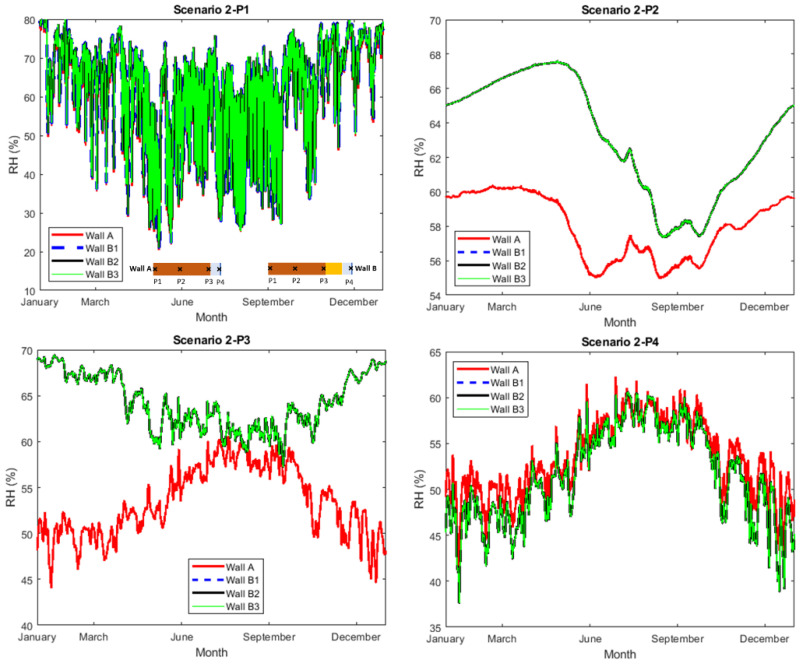
Scenario 2: Relative humidity at checkpoints P1–P4.

**Figure A6:**
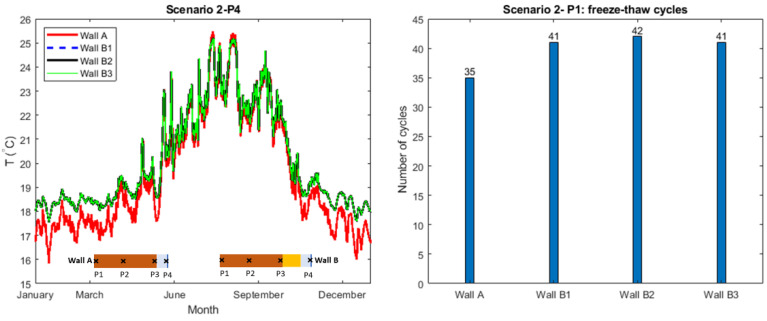
Scenario 2: Temperature at checkpoints P4 (interior surface) and number of freeze-thaw cycles at checkpoint P1 (exterior surface).

**Figure A7:**
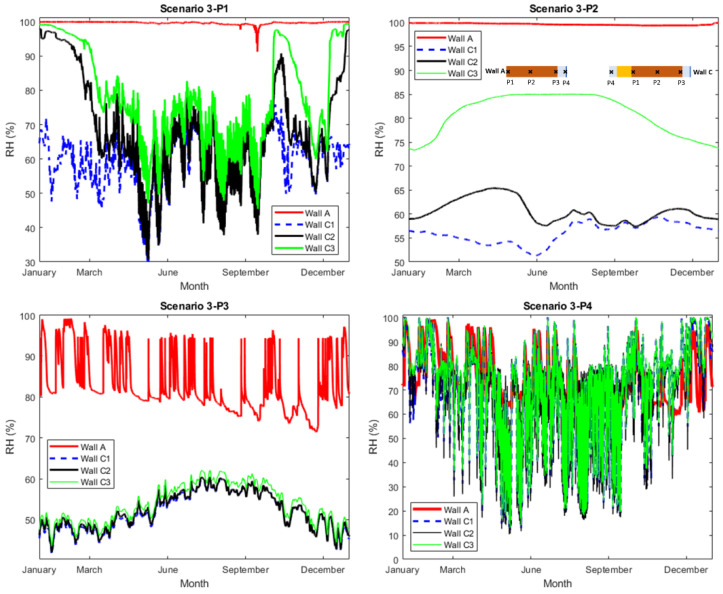
Scenario 3: Relative humidity at checkpoints P1–P4.

In Table 6, the properties of the materials used in the simulations are presented. Also, the moisture-dependent thermal conductivity of the ACM is illustrated in Figure 11. Because the specific material properties of the brick (machine-made red brick) in the reference building were unknown, the study used available brick types in the WUFI database. Based on the results of the pre-study (scenario 0) summarized in Appendix A (Figure A1 and Figure A2), the extruded brick was selected. For ACM, internal and external coating mortars and paint, the declared material properties of the products were used. For consistency, the same materials were used in all three walls (wall A, B, C). As illustrated in Figure 11, the thermal conductivity of the ACM is increased by less than 13% up to 80% RH. Above this limit, the thermal conductivity is increased by more than two times from 40 to 100 mW/(m·K).

To evaluate the impact of varying capillary water absorptivity of ACM on the hygrothermal performance of the studied walls, three different ACMs were defined as input data in the analyses. The A_cap_ for these ACMs, ACM-1, ACM-2, ACM-3, were set to 0.04, 0.2, and 0.3 kg/m^2^·min^2^, respectively. These values were chosen to based on the measured values presented in Section 4.1. Based on the selected ACM, three different wall types were considered for wall B (B1, B2, B3) and wall C (C1, C2, C3), of which wall B1 and C1 included ACM-1. Similarly, ACM-2 and ACM-3 were considered in walls B2, C2 and B3, C3, respectively.

In total, three scenarios were specified, see Table 7. For each scenario, the RH at the specified checkpoints was studied. Also, surface temperature and risk for occurrence of freeze-thawing were included in the evaluation. A freeze-thaw cycle was defined as one crossing of 0 °C in the respective checkpoint. In scenario 1 and 3, the AFR was set to 0.7, while in scenario 2, it was set to 0. The latter would represent a theoretical case where the exterior surface of the façade would undergo a water-repellent treatment to fully prevent the rainwater intrusion in the wall. It would also assume the absence of any other damage, cracks, or leakages on the surface. As previously mentioned, an external retrofitting of the reference wall is not an alternative in practice. Thus, the simulations in scenario 3 (wall C1–C3) are merely used to evaluate the impact of varying A_cap_ of the ACM on the RH levels in the walls when it is applied externally.

In Table 8, the boundary conditions for the reference case are presented. The interior climate was defined according to ISO EN 15026 [36], and based on the outdoor climate in Gothenburg, see Table 9.

## Figures and Tables

**Table 1:** The average simulated RH (%) at the checkpoints P1-P4 in different walls, scenario 1.

	P1	P2	P3	P4
Wall A	99	99	83	74
Wall B1	99	99	99	59
Wall B2	99	99	99	73
Wall B3	99	99	99	72
Maximum variation B1–B3 (%)	0	0	0	21

**Table 2:** The average simulated RH (%) at the checkpoints in different walls, scenario 2.

	P1	P2	P3	P4
Wall A	62	58	53	53
Wall B1	62	63	64	52
Wall B2	63	63	64	52
Wall B3	62	63	64	52
Maximum variation B1–B3 (%)	2	0	0	0

**Table 3:** The average simulated RH (%) at the checkpoints in different walls, scenario 3.

	P1	P2	P3	P4
Wall A	99	99	83	74
Wall B1	57	56	52	71
Wall B2	69	61	52	71
Wall B3	78	81	54	72
Maximum variation B1–B3 (%)	31	36	4	1

**Table 4:** Declared material properties of the selected commercial ACM [30] used for the measurements.

Property	Unit	Declared Value
Bulk Density (ρ)	(kg/m^3^)	180
Thermal conductivity (λ)	mW/(m·K)	40
Water vapor permeability coefficient (µ-value)	-	≤5
Water absorption coefficient (A_cap_)	kg/(m^2^·min^0.5^)	≤0.2 (W2)
Compressive strength (σ_c_)	N/mm^2^	0.5 (CS I)
Dynamic modulus of elasticity (E_dyn_)	N/mm^2^	<100

**Table 5:** The sample details and testing conditions implemented in this study.

Sample Set	Number of Samples	Sample Size	Drying Condition	Edge Condition	Number of Testing Rounds/Duration
1	3	100 × 100 × 100 mm^3^	60 °C	Sealed	3 rounds/90 min
2	3	100 × 100 × 100 mm^3^	60 °C	unsealed	3 rounds/90 min
3	3	100 × 100 × 100 mm^3^	20 °C (50% RH)	Sealed	3 ^a^ rounds/90 min
4	3	100 × 100 × 100 mm^3^	20 °C (50% RH)	unsealed	3 rounds/90 min

^a^: An additional four rounds of measurement was carried out for two out of three samples in sample set 3.

**Table 6:** Properties of the materials used in the simulations.

Material	ρ (kg/m^3^)	$\lambda_{10.\mathrm{dry}\;}$ (mW/(m·K))	µ (−)	$D_{ws.80\;}$ 10^−11^ (m^2^/s)	$D_{ws.saturation}$ 10^−8^ (m^2^/s)
Extruded brick ^a^	1650	600	9.5	5.3	4.4
Additional vapor resistance ^a,b^ (sd:0.1 m)	-	-	1000	-	-
Internal coating mortar	1200	820	10	4.1	1.0
Paint ^c^ (sd:0.01)	-	-	50	-	-
ACM-1	181	40	5	0.05	0.04
ACM-2	181	40	5	1.2	1.1
ACM-3	181	40	5	5.2	4.4

ρ: density; *λ*_10_._dry_: thermal conductivity at dry stage and 10 °C; µ: water vapor permeability coefficient; $D_{ws.80}$: liquid water transport coefficient at 80% RH; $D_{ws.saturation}$: liquid water transport coefficient at saturation. ^a^: Data extracted from the data base of WUFI. ^b^: Specified as an additional sd-value on the exterior surface of the walls. ^c^: Specified as an additional sd-value on the interior surface.

**Table 7:** Details of the scenarios included in the analyses.

Scenario	Rain: AFR	Wall A	Wall B (B1, B2, B3)	Wall C (C1, C2, C3)	Additional Vapor Resistance: Exterior	ACM
					Sd = 0.1 m	ACM-1
Scenario 1	0.7	Yes	Yes	No	Sd = 0.1 m	ACM-2
					Sd = 0.1 m	ACM-3
					Sd = 0.1 m	ACM-1
Scenario 2	0	Yes	Yes	No	Sd = 0.1 m	ACM-2
					Sd = 0.1 m	ACM-3
					Sd = 0.1 m ^a^	ACM-1
Scenario 3	0.7	Yes	No	Yes	Sd = 0.1 m ^a^	ACM-2
					Sd = 0.1 m ^a^	ACM-3

^a^: Only for Wall A.

**Table 8:** The boundary conditions specified in the simulations.

Exterior heat transfer coefficient	25 W/m^2^·K
Interior heat transfer coefficient	8 W/m^2^·K
Initial condition	8.8 °C, 74% RH ^a^
Short-wave radiation absorptivity	0.68
Long-wave radiation emissivity	0.9
Orientation	South ^b^
Adhering fraction of rain	0.7, 0
Indoor climate	ISO EN 15026: Normal moisture load

^a^: Average temperature and relative humidity in Gothenburg, see Table 9. ^b^: Most exposed direction in driving rain.

**Table 9:** The climate conditions of Gothenburg where the reference building is situated. Data is extracted from WUFI [23].

Maximum temperature (°C)	27.8	Maximum relative humidity (%)	94
Average temperature (°C)	8.8	Average relative humidity (%)	74
Minimum temperature (°C)	−12.2	Minimum relative humidity (%)	19
Average wind ^a^ (m/s)	2.97	Accumulated rain load (mm/year)	1074

^a^: Dominant wind direction: south-southwest.

## Data Availability

Data are contained within the article. Additional support can be provided by the authors upon request.

## Appendix A

In Appendix A, the results of the pre-study (Scenario 0) are presented. The pre-study was carried out to compare the hygrothermal performance of the reference wall (wall A) with respect to the brick type selected in WUFI. In Table A1 and Table A2, the details and properties of the materials used in scenario 0 are presented, respectively. To find the proper initial conditions in the wall, five consecutive years were simulated, while results are presented for the last year only. In Figure A1, the relative humidity (RH) at all checkpoints (P1–P4) is presented. Additionally, the number of freeze-thaw cycles and the total water content (W_tot_) in the brick section of the wall A are shown in Figure A2 for the last year. A freeze-thaw cycle was defined as one crossing of 0 °C in the respective checkpoint.

**Table A1:** Details of the scenario 0 (wall A) for the selection of brick type in WUFI.

Scenario	Rain: AFR	Additional Vapor Resistance: Exterior	Brick Type	ACM
		No	Masonry	NO
Scenario 0	0.7	No	Extruded	NO
		No	Historical	NO

**Table A2:** Properties of the materials used in the simulations for the pre-study, scenario 0.

Material	ρ (kg/m^3^)	$\lambda_{10.\mathrm{dry}\;}$ (mW/(m·K))	µ (−)	$D_{ws.80\;}$ 10^−11^ (m^2^/s)	$D_{ws.saturation}\;$ 10^−8^ (m^2^/s)
Brick masonry ^a^	1900	600	10	2.5	1.2
Extruded brick ^a^	1650	600	9.5	5.3	4.4
Historical brick ^a^	1800	600	15	10	9.3
Internal coating mortar	1200	820	10	4.1	1.0
Paint ^c^ (sd:0.01)	-	-	50	-	-

ρ: density; *λ*_10.dry_: thermal conductivity at dry stage and 10 °C; µ: water vapor permeability coefficient; $D_{ws.80}$: liquid water transport coefficient at 80% RH; $D_{ws.saturation}$: liquid water transport coefficient at saturation. ^a^: Data extracted from the data base of WUFI. ^c^: Specified as an additional Sd-value on the interior surface.

Based on the results of the pre-study, the studied walls with a different choice of brick type showed generally close performance considering the RH-levels at the studied checkpoints. On the exterior of the brick, P1, the average RH varied between 71–74% depending on the selected brick wall. Similarly, at checkpoints P2, P3, and P4, the variation of average RH was around 96–99, 68–68, and 59.1–59.8%, respectively. However, the RH in the wall with extruded brick was, for the majority of the year, higher than the other two walls.

The average W_tot_ in the brick masonry (saturation content: 190 kg/m^3^) was around 71 kg/m^3^ while the same for the extruded brick (saturation content: 370 kg/m^3^) and the historical brick (saturation content: 230 kg/m^3^) was 77 and 27 kg/m^3^, respectively. In other words, the water content in the brick masonry, extruded brick, and historical brick was around 37, 30, and 11% of the saturated water content, respectively. The number of freeze-thaw cycles varied between 34–37 times at checkpoint P1 during the last year of simulations. No freeze-thaw cycles occurred at checkpoints P2–P4.

The results showed that the brick masonry and extruded brick could be considered as the least favorable brick alternative, considering the water content in the brick and RH at the checkpoints. As the RH levels were higher for the extruded brick, it was selected to be used in the main study in this paper (scenario 1–3).

## Appendix B

Appendix B includes complementary figures on the results of the numerical simulations for scenario 1. In scenario 1, the exterior wall surfaces were exposed to rain (Adhering Fraction of Rain, AFR: 0.7) and drying towards exterior was highly limited, simulating a case for the locally denser and low permeable bricks in the reference building. To evaluate the impact of varying capillary water absorptivity of ACM on the hygrothermal performance of the studied walls, three different ACMs were defined as input data in the analyses. Wall A represented the original wall of the reference building prior to the retrofitting. Wall B1, B2, and B3 represented the internally retrofitted wall with ACM, at which the A_cap_ of the ACM was set to 0.04, 0.2 and 0.3 kg/m^2^·min^2^, respectively. These values were chosen based on the measured values presented in Section 4.1.

## Appendix C

Appendix C includes complementary figures on the results of the numerical simulations for scenario 2. In scenario 2, the exterior wall surfaces were not exposed to rain (AFR: 0) and drying towards the exterior was highly limited, simulating a case for the locally denser and low permeable bricks in the reference building. Wall A represented the original wall of the reference building prior to the retrofitting. Wall B1, B2 and B3 represented the internally retrofitted wall with ACM, for which the A_cap_ of the ACM was set to 0.04, 0.2 and 0.3 kg/m^2^·min^2^, respectively.

## Appendix D

Appendix D includes complementary figures on the results of the numerical simulations for scenario 3. In scenario 3, the exterior wall surfaces were exposed to rain (AFR: 0.7) and drying towards the exterior was highly limited, simulating a case for the locally denser and low permeable bricks in the reference building. Wall A represented the original wall of the reference building prior to the retrofitting. Wall C1, C2 and C3 represented the externally retrofitted wall with ACM, at which the A_cap_ of the ACM was set to 0.04, 0.2, and 0.3 kg/m^2^·min^2^, respectively.

## References

[1] Karim A.N., Johansson P., Kalagasidis A.S. (2021). Knowledge gaps regarding the hygrothermal and long-term performance of aerogel-based coating mortars. Constr. Build. Mater..

[2] Stahl T., Wakili K.G., Heiduk E. (2021). Stability Relevant Properties of an SiO_2_ Aerogel-Based Rendering and Its Application on Buildings. Sustainability.

[3] Stahl T., Wakili K.G., Hartmeier S., Franov E., Niederberger W., Zimmermann M. (2017). Temperature and moisture evolution beneath an aerogel based rendering applied to a historic building. J. Build. Eng..

[4] Fenoglio E., Fantucci S., Perino M., Serra V., Dutto M., Marino V. (2020). Energy retrofit of residential buildings with a novel super-insulating aerogel-based plaster. AiCARR J..

[5] Frick J., Sakiyama N.R.M., Stipetic M., Garrecht H. Large Scale Laboratory and Field Tests of Aerogel Renders. Proceedings of the XV International Conference on Durability of Building Materials and Components—DBMC 2020.

[6] Sakiyama N.R.M., Frick J., Stipetic M., Oertel T., Garrecht H. (2021). Hygrothermal performance of a new aerogel-based insulating render through weathering: Impact on building energy efficiency. Build. Environ..

[7] Maia J., Pedroso M., Ramos N.M.M., Flores-Colen I., Pereira P.F., Silva L. (2021). Durability of a New Thermal Aerogel-Based Rendering System under Distinct Accelerated Aging Conditions. Materials.

[8] Karim A.N. (2021). Aerogel-Based Plasters for Renovation of Buildings in Sweden: Identification of Possibilities, Information Deficiencies and Challenges. Licentiate Thesis.

[9] Cornick S.M., Lacasse M.A. (2005). A Review of Climate Loads Relevant to Assessing the Watertightness Performance of Walls, Windows, and Wall-Window Interfaces. J. ASTM Int..

[10] Geving S., Erichsen T.H., Nore K., Time B. (2006). Hygrothermal Conditions in Wooden Claddings—Test House Measurements.

[11] Lacasse M. (2003). Recent Studies on the Control of Rain Penetration in Exterior Wood-Frame Walls.

[12] Samuelson I., Mjörnell K., Jansson A. (2008). Moisture damage in rendered, undrained, well insulated stud walls. Third International Symposium on Tunnel Safety.

[13] Lacasse M.A., Miyauchi H., Hiemstra J., Wolf A.T., Dean S.W. (2009). Water Penetration of Cladding Components—Results from Laboratory Tests on Simulated Sealed Vertical and Horizontal Joints of Wall Cladding. J. ASTM Int..

[14] Kottek M., Grieser J., Beck C., Rudolf B., Rubel F. (2006). World map of the Köppen-Geiger climate classification updated. Meteorol. Z..

[15] (2016). Specification for Mortar for Masonry—Part 1: Rendering and Plastering Mortar.

[16] (2002). Methods of Test for Mortar for Masonry—Part 18: Determination of Water Absorption Coefficient due to Capillary Action of Hardened Mortar.

[17] De Fátima Júlio M., Soares A., Ilharco L.M., Flores-Colen I., de Brito J. (2016). Aerogel-based renders with lightweight aggregates: Correlation between molecular/pore structure and performance. Constr. Build. Mater..

[18] Pedroso M., Flores-Colen I., Silvestre J.D., Gomes M.G., Silva L., Sequeira P., de Brito J. (2020). Characterisation of a multilayer external wall thermal insulation system. Application in a Mediterranean climate. J. Build. Eng..

[19] Soares A., De Fátima Júlio M., Flores-Colen I., Ilharco L.M., De Brito J. (2018). EN 998-1 performance requirements for thermal aerogel-based renders. Constr. Build. Mater..

[20] Flores-Colen I., Pedroso M., Soares A., Gomes M.D.G., Ramos N.M., Maia J., Sousa R., Sousa H., Silva L. In-Situ Tests on Silica Aerogel-Based Rendering Walls. Proceedings of the XV International Conference on Durability of Building Materials and Components—DBMC 2020.

[21] Maia J., Pedroso M., Ramos N.M.M., Pereira P.F., Flores-Colen I., Gomes M.G., Silva L. (2021). Hygrothermal performance of a new thermal aerogel-based render under distinct climatic conditions. Energy Build..

[22] Pedroso M., Flores-Colen I., Silvestre J., Gomes M., Silva L., Ilharco L. (2020). Physical, mechanical, and microstructural characterisation of an innovative thermal insulating render incorporating silica aerogel. Energy Build..

[23] Ibrahim M., Wurtz E., Biwole P.H., Achard P., Sallee H. (2014). Hygrothermal performance of exterior walls covered with aerogel-based insulating rendering. Energy Build..

[24] Berardi U., Nosrati R.H. (2018). Long-term thermal conductivity of aerogel-enhanced insulating materials under different laboratory aging conditions. Energy.

[25] Nosrati R., Berardi U. (2017). Long-term performance of aerogel-enhanced materials. Energy Procedia.

[26] Morgado A., Soares A., Flores-Colen I., Veiga M., Gomes M. (2021). Durability of Thermal Renders with Lightweight and Thermal Insulating Aggregates: Regranulated Expanded Cork, Silica Aerogel and Expanded Polystyrene. Gels.

[27] Chen Y., Tang L., Gao J., Punkki J. (2018). The Surface Moisture Transport Model for Cement Mortar Under Dry-Wet Cycles. Electron J..

[28] Zhou X., Carmeliet J., Derome D. (2018). Influence of envelope properties on interior insulation solutions for masonry walls. Build. Environ..

[29] Guizzardi M., Carmeliet J., Derome D. (2015). Risk analysis of biodeterioration of wooden beams embedded in internally insulated masonry walls. Constr. Build. Mater..

[30] Wall Systems HECK AERO iP 2022. https://www.wall-systems.com/produkte/daemmputze-innendaemmung/aero-ip.

[31] METTLER TOLEDO PG503-S DeltaRange—Overview—METTLER TOLEDO n.d. https://www.mt.com/us/en/home/phased_out_products/others/PG503-S_DR.html.

[32] Balksten K., Lange J., Lindholm M. (2012). Fuktproblem i Salt-Och Frostskadat Tegelmurverk-Fördjupad Analys av Örgryte nya Kyrka.

[33] Fraunhofer IBP WUFI 2020. https://wufi.de/en/.

[34] Künzel H.M. (1995). Simultaneous Heat and Moisture Transport in Building Components: One- and Two-Dimensional Calculation Using Simple Parameters.

[35] Geving S., Holme J. (2013). Vapour retarders in wood frame walls and their effect on the drying capability. Front. Archit. Res..

[36] (2007). Hygrothermal Performance of Building Components and Building Elements—Assessment of Moisture Transfer by Numerical Simulation.

